# Consumer behavior and sustainability: Exploring Italy's green cosmetics market with prickly pear seed oil

**DOI:** 10.1016/j.heliyon.2025.e42233

**Published:** 2025-01-25

**Authors:** Giuseppe Timpanaro, Giulio Cascone

**Affiliations:** Department of Agriculture, Food and Environment, University of Catania, Via S. Sofia 100, 95123, Catania, Italy

**Keywords:** Natural cosmetics, Environmental impact, Sustainability, Eco-friendly technologies, Consumer awareness

## Abstract

Prickly pear seed oil is becoming increasingly popular in green cosmetics due to its skin benefits. However, there is a lack of research on consumer demand and perception of products containing this ingredient in Italy. This study aims to address this gap by examining the factors that influence Italian consumers' demand for prickly pear seed oil. Specifically, we focus on the product's attributes, as well as socio-demographic factors. The research engaged 300 Italian participants through an online survey. The dataset was analyzed using an Ordered Probit Model, revealing a higher purchasing frequency for prickly pear seed oil among consumers who prioritize the product's natural and sustainable origins. The analysis also highlights the influence of socio-demographic characteristics, particularly gender and income, on purchasing behavior. These findings provide valuable insights for marketers and product developers in the green cosmetics industry. Emphasising the natural and sustainable aspects of prickly pear seed oil can appeal to a broader audience. Additionally, understanding the demographic factors at play can help tailor marketing strategies to target specific consumer segments more effectively. The study contributes to the strategic development of marketing and product innovation in the green cosmetics sector by uncovering consumer preferences and trends. It advocates for a deeper consumer-centric approach in promoting sustainable beauty products.

## Highlights

• Prickly pear seed oil is among the oldest natural cosmetics.
• Factor analysis, clustering, and the ordered probit model revealed key insights into prickly pear seed oil consumption.
• The purchase of prickly pear seed oil is influenced by environmental impact, safety, and label information.
• 'Selective', 'Sustainable', and 'Pragmatic' consumers tend to buy prickly pear seed oil more frequently.
• Female gender and high income significantly influence the purchase of prickly pear seed oil.

## Graphical abstract

**Figure:**
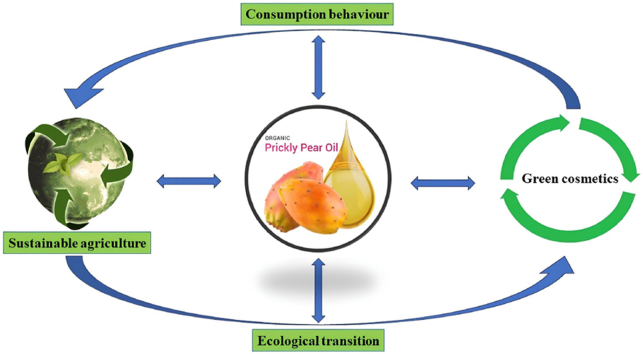

## 1 Introduction

The demand for cosmetic products made with natural ingredients is on the rise due to the growing focus on environmental sustainability and responsible production practices. Consumers are increasingly valuing sustainability and well-being. This type of consumption promotes the use of renewable resources, waste reduction, ethical business practices, innovation, and the development of more eco-friendly technologies. This contributes to the transition towards a more sustainable economy [[1], [2], [3], [4]]. Consequently, the term 'green cosmetics' has been coined to refer to products formulated with natural, organic, and sustainable ingredients, often avoiding chemicals that are harmful to both the environment and human health. The consumption of these products supports ethical production practices, such as responsible sourcing of ingredients and the use of eco-friendly packaging [5]. Furthermore, they promote consumer awareness regarding the importance of reducing the environmental impact of their consumption choices, encouraging a lifestyle more attuned to personal and environmental well-being [6,7].

Plant-based cosmetic oils are a common ingredient in green cosmetics products due to their numerous benefits for the skin and their sustainability compared to oils derived from non-renewable sources [[8], [9], [10]].

Green cosmetics have gained increasing attention in recent years, both from the academic community and industry professionals.

A recent review [11] provided a comprehensive explanation of consumer decision-making through a vision using the SOR paradigm, identifying and ranking the main environmental signals that encourage or limit their choices for organic cosmetics. The results of the review highlighted the need for a consistent, transparent and clearly established regulatory or licensing system for organic cosmetics. The absence of clear and universal regulation also encourages the emergence of misleading marketing practices, such as ‘greenwashing’, where products are presented as environmentally sustainable or organic without clear and verifiable environmental benefits. Ambiguous labels and misleading claims about the origin of ingredients or production processes make consumers sceptical, weakening the green cosmetics market. A uniform and internationally recognised certification standard could not only reduce this confusion, but also increase the transparency and reliability of green products, strengthening consumer confidence. In this way, customers would be more likely to make informed choices, based on a genuine knowledge of the benefits and impact of the products they buy.

Scientific literature has amply demonstrated that psychological factors, such as attitudes towards green cosmetics, play a decisive role in consumers' purchase intentions [[12], [13], [14], [15]]. In addition to attitudes, values related to environmental sustainability prove to be crucial in purchasing decisions [13,16], as well as awareness of and concern for environmental issues [17,18]. Social and subjective norms have also been shown to be influential in shaping purchasing behaviour [12,14], along with the consumer's own perception of control [[12], [13], [14]]. Furthermore, several studies show that ethical concern, perceived functional value, and attributes of eco-friendly products are among the predictors of purchase intention. These attributes include fair trade, cruelty-free and green formulation, eco-labeling, and eco-friendly packaging [[19], [20], [21]].

Other key elements that impact consumers' choices regarding green cosmetics include health-related awareness and the desire to promote a healthy lifestyle [17,18,22,23]. Factors such as product innovation [23] and trust, particularly in the safety of cosmetics, also play an important role in influencing purchasing decisions [15]. Finally, a study by Ref. [24] found that attitudes and perceptions of brand value have a significant impact on Chinese consumers' intentions to purchase green cosmetics. Branding increases perceptions of product quality and positively affects consumers' choice to buy green cosmetics, offering a guarantee to consumers who opt for sustainable products [25]. Available research on the labelling and promotion of natural cosmetics suggests that green product labels play a significant role in consumers' purchase intentions [26], while eco-friendly advertising has been shown to positively influence attitudes towards luxury organic cosmetics [15]. [27] pointed out, among other factors, that well-crafted promotion and ‘visual appeal and physical signs at the point of sale’ were crucial in driving purchase behaviour [28]. observed that the frequent use of naturalness claims on personal care product packaging had a positive impact on consumers' purchase intentions.

Prickly pear seed oil has become increasingly popular in the green cosmetic industry due to its ability to provide numerous skin benefits. It has excellent moisturizing, anti-aging, and antioxidant properties that can enhance skin elasticity and minimize redness and discoloration [29,30]. Additionally, its balance of linoleic acid to oleic acid, at a ratio of 3:1, makes it ideal for cosmetic use [31]. The oil's antimicrobial properties make it valuable for developing skin treatments, including those for acne. Additionally, research has shown that the oil contains vitamin K1 (0.53 g/kg), which can help reduce the appearance of dark circles and varicose veins [32]. In addition to direct skin application, this oil is used as a base for other cosmetic and hair products, expanding the range of natural beauty solutions available [33].

Prickly pear seed oil is a sustainable option for green cosmetics, as it promotes more ecological and responsible practices that help mitigate climate change [[34], [35], [36], [37]]. The use of natural and sustainable ingredients is the focus of green cosmetic products. This helps to reduce the environmental impact of cosmetic production and use. Prickly pear seed oil is an example of a sustainable ingredient, as it is extracted from a resilient plant that requires minimal water resources to grow. This aligns with the philosophy of sustainable cosmetic products [38,39]. Furthermore, the cosmetic industry has the potential to contribute to mitigating climate change by reducing the use of environmentally harmful ingredients, decreasing greenhouse gas emissions during production, and adopting biodegradable packaging. For instance, the use of natural ingredients such as prickly pear seed oil has been shown to significantly reduce the overall environmental impact of the cosmetic industry [[40], [41], [42], [43]]. Sustainability is crucial in addressing climate change. Therefore, the use of green cosmetic products and ingredients, such as prickly pear seed oil, can help reduce the individual and collective ecological footprint. This, in turn, supports more sustainable practices in the cosmetic industry and beyond. Several studies have shown the benefits of using such products [[44], [45], [46], [47]].

Despite the growing interest in green cosmetics and the growing literature on this topic, few studies have dealt in depth with the segmentation of consumers according to specific product attributes. Most existing research focuses on general aspects of sustainable consumption or attitudes towards natural cosmetics, but there is a lack of detailed analysis exploring how different consumer segments value specific attributes. This study attempts to fill this gap by exploring consumer preferences towards prickly pear seed oil, a niche product in the green cosmetics sector, with a specific focus on consumer segmentation based on perceived product attributes. The novel element of this work lies in the use of a segmentation methodology based on consumer attitudes and lifestyles, going beyond the mere analysis of purchase intentions. This approach makes it possible to identify homogeneous clusters of consumers sharing similar preferences, offering a deeper understanding of the green cosmetics market and the dynamics influencing the demand for specific products such as prickly pear seed oil. In addition, frequency of purchase was chosen as the dependent variable instead of quantity purchased, another element that distinguishes this study from the existing literature, as it allows for a more accurate capture of recurring consumption habits and the factors that determine constant demand over time. Unlike similar studies, we use purchase frequency as the dependent variable instead of product quantity. Previous studies have solely concentrated on the consumption behavior of the prickly pear fruit [[48], [49], [50]] or its use as an ingredient in functional foods [51,52]. However, none have explored the extracted oil, which represents a novel area of research. The objective of this study is to determine the main factors that influence the demand for prickly pear seed oil and their role in this context. To achieve this, we meticulously examined various attributes to categorise homogeneous market segments for prickly pear oil based on consumer attitudes and lifestyles. To accomplish this objective, the following research question was identified.

RQ1 What are the most significant attributes for consumers of prickly pear seed oil?

RQ2 Can consumers be categorized into distinct groups based on their preferences for these features?

RQ3 Which market segment tends to buy prickly pear seed oil more often?

RQ4 How do socio-demographic characteristics of consumers affect their buying habits and preferences for prickly pear seed oil?

## 2 Materials and methods

**Fig. 1:**
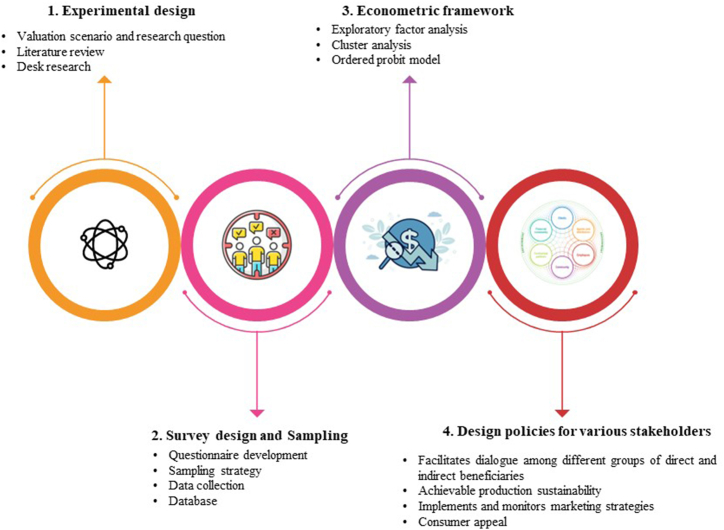
The proposed research model.

The research was conducted according to the structure proposed in Fig. 1. First, a desk survey of the research conducted to date, secondary data and existing literature on the subject was carried out, with the aim of achieving a clear identification of the research questions. This was followed by a phase of constructing a survey questionnaire and defining the sample, its size and representativeness. Subsequently, the primary data were collected and the most appropriate methodologies defined for their econometric processing. Finally, the results were analyzed from the perspective of offering a useful information base to various categories of stakeholders interested in cacti derivatives.

### 2.1 Selection of key attributes

In this study, we adopted a scale inspired by the Food Value Scale (FVS), developed by Ref. [53], which is widely used in agricultural economics to explore personal values related to food choices. However, considering that our context concerns green cosmetics, we adapted this scale to reflect the specific factors that influence purchasing decisions in this sector. The factors were chosen based on the existing literature and the distinctive characteristics of natural cosmetics.

The factors examined in our study include: naturalness (the degree to which the product is free of artificial chemicals), environmental impact (the effect of the production and use of the cosmetic on the environment), label information (the transparency and clarity of the information provided about the product), branding (the trust placed in the cosmetic brand), container volume (the amount of product available), appearance (the attractiveness of the packaging and the product), user-friendliness (ease and comfort in using the cosmetic), price (the cost of the product), safety (ensuring that the product is safe for skin and health), and origin (the origin of the ingredients used). Previous studies have explored food values as a tool to identify stable constructs in consumer preferences [54,55].

These factors reflect a combination of classic Food Value Scale items adapted to the green cosmetics sector, with the addition of specific attributes such as label information, brand, and container volume, which are particularly relevant in the context of natural cosmetics. The selection of these factors is justified by the need to analyse consumer perceptions of green cosmetics in detail, considering personal values and expectations of transparency, quality and sustainability.

Label information plays a key role in guiding consumer choice. Transparent and eco-friendly labels increase the positive perception of green cosmetics, making it easier to understand their quality and the environmental and health standards they adhere to. However, the lack of uniform regulation in the natural cosmetics sector can lead to confusion among consumers, as different certification bodies adopt varying criteria for the admission of natural or organic ingredients [56,57]. Therefore, clear and well-defined labelling is a crucial element in improving trust and reducing scepticism towards these products [58].

Branding is a decisive factor that positively influences consumers' perceptions of the quality of green cosmetics and purchase choices [59,60]. Brands offer fundamental reassurance to consumers who prefer environmentally friendly products, guaranteeing the reliability and authenticity of products, and thus reducing doubts related to quality or production practices [24,25]. This trust is reinforced by the credibility image associated with the most established brands, which helps reduce the perceived risk in the purchasing process. Brand loyalty and positive image also play a crucial role in consumer decision-making, stimulating greater attachment and propensity to choose green cosmetics from well-known brands [20]. Consequently, established brands can act as guarantors of quality and sustainability, positioning themselves as key elements in marketing strategies for green cosmetics.

Although it has not been extensively studied in the literature, the volume of the container is a critical factor for consumers of cosmetics, as it can influence both the perception of value and the user-friendliness of the product. Consumers tend to appreciate larger packages, as these convey a feeling of greater durability or cost-effectiveness, contributing to a perception of higher overall product value. At the same time, other consumers may prefer smaller formats, considering them more practical and easier to carry, especially in travel contexts or for more convenient everyday use [61]. The choice of packaging is therefore relevant not only for the content, but also for the overall image that the product communicates to the customer. This preference for appropriate packaging is not only limited to the cosmetics sector, but is also common in other markets, where the size of the container plays a decisive role in purchasing decisions, influencing the perceived usefulness and convenience of the product [62].

### 2.2 Data collection

Data were collected by distributing a survey through Google Forms (Appendix A) and sharing it on social networks and specialised forums. This approach was chosen because it allowed for a broad and diverse respondent base, overcoming geographical barriers [63]. The survey was conducted from January 2023 to August 2023 to capture potential consumers interested in seed oil for addressing cosmetic issues related to harsh weather conditions. The completion of the questionnaire implied informed consent to participate in the study, and participants were provided with details about the research purpose prior to participation. A total of 300 consumers responded to the survey, which consisted of three parts. The sample size is in line with many studies on consumer behaviour in the green cosmetics market [11], due to the niche nature of the market and the costs associated with data collection. In this regard, it is also possible to recall general studies on agrifood [64,65]. In the first part of the questionnaire, we provided the respondents with a detailed description of prickly pear seed oil to ensure that they were aware of the type of product proposed. Subsequently, after a screening question on awareness of prickly pear oil, respondents rated their interest in specific product attributes on a 5-point Likert scale (ranging from 1, not important, to 5, very important). The second part aimed to determine the most common purchasing locations for prickly seed oil, including online platforms, specialised shops, pharmacies, and perfumeries. This information highlights the accessibility and preferred purchasing locations for the product. Additionally, the study investigated consumers' preferences for different commercial formulations of prickly seed oil, such as pure oil, creams, serums, or as an ingredient in other cosmetic products. It also examined the colour preference for the oil, which may vary depending on the extraction method and the specific prickly pear variety used. The survey investigated consumer preferences for the production technology of prickly pear seed oil, such as cold extraction, refining, or fruit maceration methods. The last part collected sociodemographic information, including gender, age, education level, area of residence, and monthly household income. Although social networks are accessible to users from around the world, participation was primarily from individuals of Italian nationality. The popularity of the prickly plant in Italy may explain such a response. Italy has over 8.6 thousand hectares of prickly plant surface area and produces almost 155 thousand tons of prickly products, with over 90 % of the production coming from Sicily [66]. To ensure the quality and clarity of the questions, a pre-test was conducted on a sample of 35 respondents, representative of the target group of consumers interested in prickly pear seed oil. During the pre-test, we administered the questionnaire and subsequently collected feedback on the questions regarding their comprehensibility, relevance and relevance. Based on the suggestions received, we reworded unclear questions and eliminated redundant ones. These changes improved the overall quality of the questionnaire, ensuring that the questions were clear and that respondents were able to provide more accurate and meaningful answers.

### 2.3 Data analysis

To synthesize the attributes of prickly pear seed oil that showed strong correlations, the statistical analysis started with an exploratory factor analysis (EFA). Using the principal component method, this technique clusters the original variables into orthogonal factor dimensions [67,68]. The determination of the number of components was carried out using the eigenvalue rule with a threshold of 1 [69]. A varimax rotation was then applied to the resulting components to facilitate clearer definitions and interpretations of the factor dimensions [70,71]. To facilitate understanding of the factors, loadings below 0.4 were excluded in the final presentation of the factor dimensions [72].

The validity of the model was assessed using the Kaiser-Meyer-Olkin (KMO) test and Bartlett's test, which are based on partial correlations between variables. The results of the KMO test vary between 0 and 1; lower values indicate that the analysis may not be appropriate, as the correlation between pairs of variables cannot be accounted for by the common variance of the entire set of variables. Therefore, it is advisable that the results of the KMO test do not fall below 0.5, while values above 0.7 are considered good [73].

Bartlett's test is frequently used to check whether the correlation matrix is equivalent to the identity matrix [74]. A non-significant result in Bartlett's test suggests that the identity matrix might coincide with the correlation matrix, thus making the use of the factorial model inappropriate.

To cluster consumers of prickly pear seed oil based on their preferences, a non-hierarchical cluster analysis was conducted using the k-means method applied to the factors identified in the initial phase. This method relies on an iterative process that minimizes the Euclidean distances between the centroids of the groups [75,76]. One limitation of non-hierarchical methods is the need to pre-determine the number of groups to create, which introduces an element of subjectivity and researcher expertise [77,78]. To determine the appropriate number of clusters, the algorithm can be executed multiple times with varying numbers of groups, and the results can be evaluated in terms of internal distances and between groups [79]. Various cluster solutions were tested and compared, and the four-cluster solution was found to be the most suitable.

To estimate the determinants of the purchase frequency of prickly pear seed oil, an Ordered Probit Model (OPM) was used. This approach was chosen due to the ordinal nature of the dependent variable, which represents the purchase frequency of prickly pear seed oil [80]. The model is described in the equation:

$$y_{i}*=xi\;\beta +e_{i}\text{,}$$

where: *xi* represents a vector of independent variables including cluster membership and sociodemographic characteristics; and *β* is the vector of unknown parameters. The dependent variable *yi*∗ is measured by four levels of purchase frequency of prickly pear seed oil (1st level: "I rarely purchase prickly pear seed oil", 2nd level: "I purchase prickly pear seed oil monthly", 3rd level: "I purchase prickly pear seed oil weekly", 4th level: "I purchase prickly pear seed oil more than once a week"). During the mapping process, the following set of alternatives for consumers was utilized:

$$y_{i}=1\;\text{if}\;\mu_{0}\;<\;y_{i}*\leq \mu_{1}\text{;}$$

$$=2\;\text{if}\;\mu_{1}\;<\;y_{i}*\leq \mu_{2}\text{;}$$

$$=3\;\text{if}\;\mu_{2}\;<\;y_{i}*\leq \mu_{4}\text{;}$$

$$=4\;\text{if}\;\mu_{3}\leq y_{i}*\text{;}$$

The OPM is used to generate data for discrete choice dependent variables [81,82]. Its main purpose is to predict the probabilities of choices based on changes in independent variables [83,84]. The estimated parameters in the OPM can be interpreted directly. A positive sign indicates that the probabilities for the alternatives shift towards higher categories as the explanatory variable increases [85]. This shift occurs while other variables in the model remain constant [86]. For the statistical analysis, the statistical software STATA 15 was used.

## 3 Results

### 3.1 Socio-economic profile of the sample

Table 1 presents the demographic data for the sample analyzed in this study. The gender distribution shows a predominance of female participants, accounting for 71 % of the sample, compared to 29 % male. In terms of age, more than half of the participants (52 %) fall within the age range of 19–36 years, indicating a significant representation of young adults. However, it is important to note that 21.67 % of the sample consists of individuals over the age of 55, indicating a diverse range of generational perspectives. Regarding education, most participants (74 %) have completed university education, indicating a high level of education within the sample. The participants' area of residence shows that 76 % of them are from Sicily, while the remaining 24 % come from other regions, providing a diverse range of geographical perspectives within the sample. Additionally, the distribution of participants by monthly household income is diverse, with 43 % earning more than €4000 per month and 19.33 % having an income below €2000. The survey responses may have been influenced by financial differences.

**Table 1:** Socio-economic characteristics of the sample (∗).

Variable name	Percentage
Gender	
Female	71 %
Male	29 %
Age	
19–36	52 %
37–54	26.33 %
>55	21.67 %
Education	
Primary	0.67 %
Secondary	1.67 %
High school	23.67 %
University	74 %
Residence Area	
Sicily	76 %
Other regions	24 %
Monthly household income	
<2000 €	19.33 %
2000–4000	37.67 %
>4000	43 %

(∗) Authors' elaboration.

As shown in Table 2, the frequency of purchasing prickly pear seed oil among the respondents varied significantly. Almost half of the respondents (49.33 %) stated that they buy the oil on a weekly basis, while 33.67 % buy it more than once a week. A smaller percentage of respondents (14.67 %) purchased the product monthly and only 2.3 % of the sample purchased it rarely. This distribution underlines the high regularity with which the respondents consume prickly pear seed oil, reinforcing the relevance of frequency of consumption as an important factor in our analysis.

**Table 2:** Frequency of Prickly Pear Seed Oil purchases among respondents (∗).

Dependent variables	Frequencies	Percentage
"I rarely purchase prickly pear seed oil"	7	2.3 %
"I purchase prickly pear seed oil monthly"	44	14.67 %
"I purchase prickly pear seed oil weekly"	148	49.33 %
"I purchase prickly pear seed oil more than once a week"	101	33.67 %

(∗) Authors' elaboration.

**Fig. 2:**
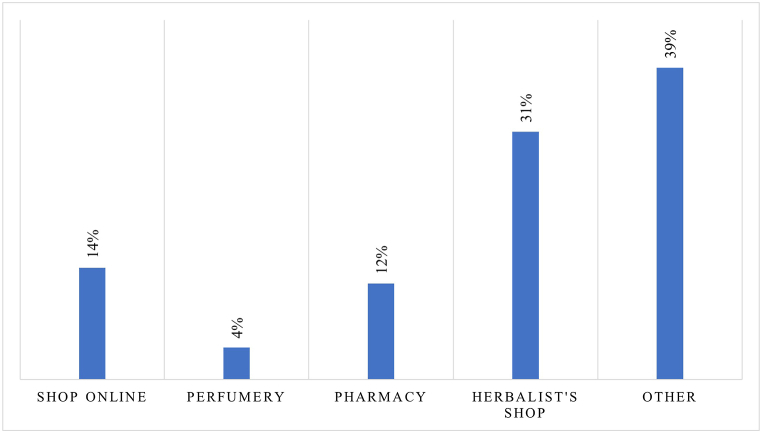
Channels of purchase of prickly pear seed oil in the consumer sample.

Table 3 presents the descriptive statistics for the attributes assessed by consumers, showing the mean and standard deviation for each on a scale of 1–5. Fig. 2 shows the structure of the purchasing channels preferred by consumers of prickly pear seed oil. On average, the herbalist's shop channel was prevalent (alone intercepting 31 % of preferences), although the role of other specialised and non-specialised shops (e.g. pharmacies, online shops) was not negligible.

**Table 3:** Summary statistics of consumers' perceived attributes of prickly pear seed oil (∗).

Variable	Obs	Mean	SD
Appearance	300	2.64	1.16
Environmental impact	300	3.84	1.05
Label information	300	3.48	1.01
Brand	300	2.85	1.05
Naturalness	300	3.22	1.17
Origin	300	2.91	1.14
Practicality of use	300	2.57	0.63
Price	300	2.88	0.83
Safety	300	3.59	1.20
Container volume	300	3.21	1.23

(∗) Authors' elaboration.

### 3.2 Exploratory factor analysis (EFA) results

Fig. 2 shows the structure of the purchasing channels preferred by consumers of prickly pear seed oil. On average, the herbalist's shop channel was prevalent (alone intercepting 31 % of preferences), although the role of other specialised and non-specialised shops (e.g. pharmacies, online shops) was not negligible.

An Exploratory Factor Analysis (EFA) was conducted on the attributes to synthesize them. The EFA results identified five principal factors, each representing a latent construct based on the factorial loadings of various variables, as shown in Table 4. Regarding the model fit, the values of the KMO test (0.656) and the Bartlett test (<0.000) are significant (Table 5).

**Table 4:** Exploratory factor analysis (EFA) performed on perceived attributes of prickly pear seed oil (∗).

	Factor 1 Practicality and Price	Factor 2 Safe and Volume	Factor 3 Brand and Appearance	Factor 4 Naturalness and Origin	Factor 5 Sustainability and Label information
Appearance			0.83		
Environmental impact					0.83
Label information					0.73
Brand			0.84		
Naturalness				0.76	
Origin				0.86	
Practicality of use	0.85				
Price	0.88				
Safety		0.89			
Container volume		0.86			

(∗) Authors' elaboration.

**Table 5:** Sampling adequacy test (KMO) and Bartlett's sphericity test on the sample of consumer (∗).

Kaiser–Meyer–Olkin's measure		0.656
Bartlett's Test of Sphericity	Approx. Chi-Square	463.716
	df	45
	Sig.	0.000
Determinant		0.207

(∗) Authors' elaboration.

**Fig. 3:**
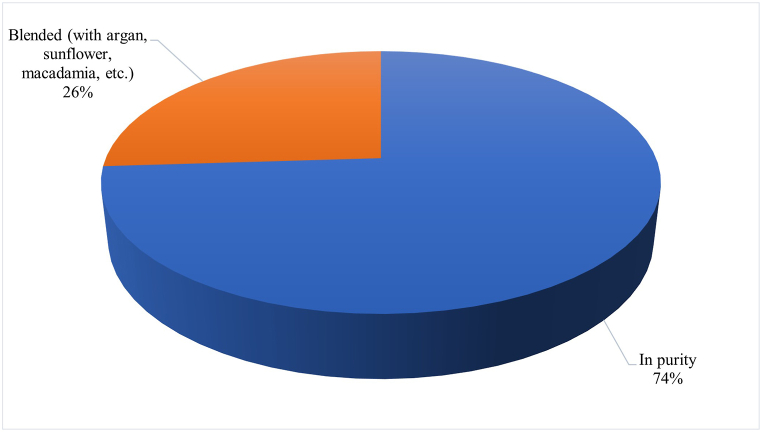
Preference of prickly pear seed oil by type of commercial formulation in the consumer sample.

**Fig. 4:**
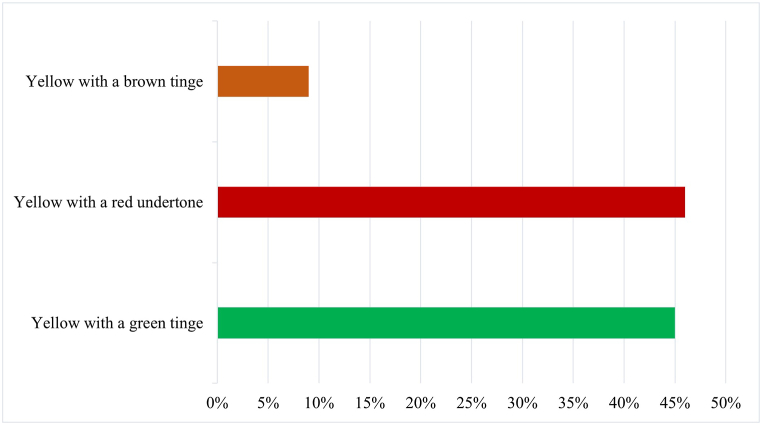
Preference of prickly pear seed oil by colour in the consumer sample.

**Fig. 5:**
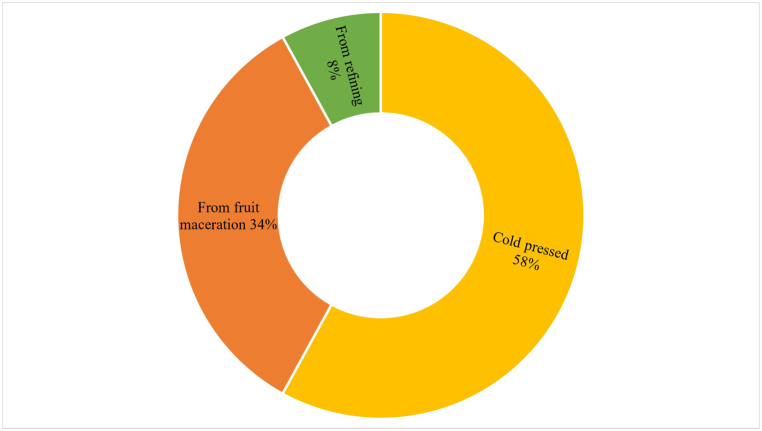
Preference of prickly pear seed oil according to production technology in the consumer sample.

The first factor, named 'Convenience and Price,' includes ease of use (0.85) and price (0.88). The results suggest a strong correlation between consumer interest in products that are easy to use and affordably priced. This suggests that consumers may prefer prickly pear seed oil options that offer a favourable quality-price ratio and convenience. The second factor, named 'Safety and Container Volume,' indicates that consumers place particular emphasis on product safety and the quantity offered. This highlights an interest in safe products provided in adequate quantities. The third factor, named 'Brand and Appearance,' is positively correlated with brand (0.84) and appearance (0.83). This factor suggests that consumers consider brand identity and the visual appeal of the product to be important. Therefore, a strong brand and attractive packaging may be key elements in the consumer decision-making process. The fourth factor, 'Naturalness and Origin,' is positively associated with the product's naturalness (0.76) and origin (0.86). This factor shows that consumers value the authenticity and origin of the product, preferring prickly pear seed oils that are perceived as natural and deeply rooted in their place of origin. Indeed, the pure product is widely preferred (as shown in Fig. 3) and yellow in colour with shades of green and red (Fig. 4). To preserve the aspects of naturalness, the well-informed consumer also expresses a preference for the extraction technology (cold pressed), as it is positively valued for its impact on the final quality of the commercial product (Fig. 5).

The last factor identified through the EFA is named 'Sustainability and Label Information' because it has a positive correlation with environmental impact (0.83) and label information (0.73). This factor emphasises the significance that consumers attach to environmental considerations and the transparency of information provided about the product. This reflects an increasing awareness of environmental issues and a desire to be informed about the characteristics and impact of the products they purchase.

### 3.3 Cluster analysis results

After conducting Exploratory Factor Analysis (EFA) to reduce the perceived attributes of prickly pear seed oil consumers, we used the resulting factors to segment consumers through cluster analysis. Factor scores made it possible to identify similarities and differences between different consumer groups, facilitating a more precise and informed segmentation. Table 6 presents the outcomes, identifying four distinct clusters. Cluster 1 (n = 74) comprises consumers who prioritize brand and appearance, naturalness and origin, but place less importance on convenience and price, and show less interest in sustainability and label information. This group can be identified as 'Selective Consumers'. Cluster 2 (n = 92), values convenience and price highly, but shows minimal concern for safety, volume, brand, appearance, naturalness, and origin, and mild indifference towards sustainability. These are the 'Pragmatic Consumers', who focus on practical and economic aspects. Cluster 3 (n = 60) highlights a strong interest in sustainability and label information, coupled with a moderate preference for brand and appearance, but scant interest in naturalness and origin. This group, named 'Sustainable Consumers', shows a clear inclination towards sustainable choices. Lastly, Cluster 4 (n = 74) is distinguished by a marked interest in safety and volume, paired with moderate attention to brand and appearance, but lesser consideration for convenience, naturalness, and sustainability. This group, named 'Cautious Consumers', emphasises the safety and quantity of the product.

**Table 6:** Cluster analysis performed on the sample of prickly pear seed oil consumers (∗).

Cluster	Practicality and Price	Safe and Volume	Brand and Appearance	Naturalness and Origin	Sustainability and Label information
Selective Consumers	−1.24	−0.09	0.19	0.24	−0.42
Pragmatic Consumers	0.52	−0.57	−0.57	0.16	−0.10
Sustainable Consumers	0.04	−0.03	0.38	−0.59	0.63
Cautious Consumers	0.26	0.83	0.21	−0.21	−0.53

(∗) Authors' elaboration.

### 3.4 Ordered probit model (OPM) results

The study analyzed the frequency of consumers purchasing prickly pear seed oil using an OPM. The results, presented in Table 7, offer a comprehensive understanding of purchasing habits. To avoid perfect collinearity issues associated with dummy variables, the fourth group was excluded from the analysis. The study shows that there is a positive and significant association between the frequency of oil purchase and the consumer groups identified as 'Selective Consumers', 'Pragmatic Consumers', and 'Sustainable Consumers'. The 'Selective' group had the highest frequency of product purchase (β = 0.640, p = 0.001), followed by 'Sustainable Consumers' (β = 0.306, p = 0.006), and finally 'Pragmatic Consumers' (β = 0.270, p = 0.070). In terms of the sociodemographic characteristics of the sample, gender appears to be a significant factor, with a positive impact on the likelihood of frequent purchases of prickly pear seed oil (β = 0.577, p = 0.001). This suggests that women are more likely to purchase the product. In addition, monthly income is a significant determinant with a positive effect on the likelihood of purchasing (β = 0.478, p = 0.001). This demonstrates that individuals with higher incomes are more likely to purchase prickly pear seed oil.

**Table 7:** Ordered probit regression on prickly pear seed oil purchase frequency (^a^).

Variable	Coeff.	Std.Err	p-value
Selective Consumers	0.640	0.21	0.001∗∗∗
Pragmatic Consumers	0.270	0.20	0.070∗
Sustainable Consumers	0.306	0.20	0.006∗∗
Cautious Consumers			
Gender	0.577	0.14	0.001∗∗∗
Age	−0.112	0.09	0.195
Education	0.004	0.13	0.976
Month Income	0.478	0.09	0.001∗∗∗
/cut1	−1.006		
/cut2	0.207		
/cut3	1.783		

H0: cut1 = cut2 p-value <0.001			
H0: cut2 = cut3 p-value <0.001			
Wald chi^2^(7) = 66.83 p-value <0.001			

∗∗∗, ∗∗, ∗ Indicate significance at the 0.01, 0.05, and 0.10 levels, respectively.

(^a^) Authors' elaboration.

## 4 Discussion

Based on the data and analyses conducted, this study addresses the research questions. Regarding the first research question, it is evident that consumers of prickly pear seed oil perceive various attributes with different degrees of preference. The most influential attributes for consumers are environmental impact (3.84), safety (3.59), and label information (3.48). The scores obtained suggest that consumers are increasingly ecologically aware and value transparency and safety. This highlights the importance of sustainable practices and informative labelling in marketing strategies [18,56,87]. In contrast, aspects such as ease of use (2.57) and appearance (2.64) received lower scores, indicating that they are less of a priority for consumers. However, these areas could be improved to increase product appeal. These insights offer a clear understanding of consumer priorities, indicating opportunities for producers and sellers of prickly pear seed oil to meet these needs. Additionally, they raise interesting questions about how consumers form their perceptions of value and quality.

Regarding the second research question, the non-hierarchical cluster analysis revealed distinct consumer categories based on their evaluations of prickly pear seed oil attributes. The first cluster, referred to as 'Selective Consumers', demonstrated a neutral attitude towards ease of use and price, but expressed interest in brand, origin, and naturalness. This may indicate that these consumers are less sensitive to price and place greater importance on the product's aesthetics and perceived naturalness [88]. highlighted that consumers choose green cosmetics not only for their ecological properties but also to satisfy beauty needs. Similarly, 'Selective Consumers' may perceive prickly pear seed oil as a product that enhances their physical appearance, associating the product's naturalness with healthier skin [28,89,90]. Furthermore, previous studies have shown that the origin of a product plays a significant role in the decision-making process when purchasing green cosmetics, often as a way of supporting local and national producers [14].

The second cluster, 'Pragmatic Consumers', values attributes such as convenience and price when purchasing prickly pear seed oil. This behaviour is consistent with observations in the field of green cosmetics, where price is often considered a decisive factor. Studies have shown that consumers may perceive green cosmetics as more expensive than conventional alternatives, which can influence their purchasing decisions [60,91]. The importance of marketing strategies that highlight the value and ease of use of prickly pear seed oil is underscored by the authors' inclination towards practical and accessible solutions [58,92].

The third cluster, 'Sustainable Consumers', is characterised by a focus on sustainability and label information. These consumers exhibit a strong level of environmental concern, which is a key factor in their purchasing decisions. Recent studies have shown that environmental concern is a significant predictor of the intention to purchase green cosmetics [5,18,93]. Labelling information is crucial for 'Sustainable Consumers', particularly in the context of sustainability. This information helps them make sustainable decisions that align with their environmental values [17,57].

Finally, the 'Cautious Consumers' prioritises safety and container volume over external features such as brand or aesthetics. Their lesser emphasis on convenience and price suggests that these consumers may be willing to invest more to ensure the product is safe and of high quality [24]. Research indicates that consumers' attitudes and acceptance towards green cosmetics are influenced by their safety perception, which is often linked to environmental concerns and ethical judgments [13,22,23,94].

The findings from the OPM (addressing the third research question) offer interesting insights into the purchasing frequency of prickly pear seed oil among different consumer segments. It is noteworthy that the purchasing frequency of prickly pear seed oil is highest among 'Selective Consumers', followed by 'Sustainable Consumers' and then 'Pragmatic Consumers'. This finding is consistent with the trends identified in the literature, where an increasing number of consumers prefer natural products with clear origins, as they are linked to higher quality and reduced environmental impact [25,95,96].

Additionally, the OPM analysis indicates that 'Sustainable Consumers' are more likely to purchase prickly pear seed oil. This group prioritises sustainability and the environmental impact of products. The choice is likely influenced by the perception of the oil as an environmentally friendly product. This reflects a growing environmental awareness and the search for products that align sustainability values with consumption choices [97]. This is consistent with previous studies that have highlighted the importance of environmental awareness in consumer choices when purchasing green cosmetics [16,98]. Finally, "Pragmatic Consumers" exhibit a lower probability of consuming prickly pear seed oil, yet it is essential to acknowledge the influence of perceived value for money in the purchase of green cosmetics, in line with a practical and value-oriented approach [99,100].

In relation to the sociodemographic characteristics (addressing the fourth research question), the analysis of the purchasing frequency of prickly pear seed oil reveals some important observations. Firstly, gender plays a significant role, with women tending to purchase prickly pear seed oil more frequently than men. This trend may be explained by a greater propensity among women to purchase products perceived as healthy or beauty-enhancing, a phenomenon observed in numerous other studies [20,[101], [102], [103]]. Additionally, it is noteworthy that higher monthly income increases the likelihood of purchasing prickly pear seed oil, suggesting that individuals with higher incomes tend to buy it more frequently. The frequency of purchasing prickly pear seed oil may be limited by its perception as a luxury or niche product, which may be too expensive for consumers with lower incomes [104,105]. Surprisingly, age and educational level do not appear to significantly impact purchasing frequency. Regardless of age or educational level, consumption patterns of prickly pear seed oil remain relatively constant. This is consistent with previous studies that have found these characteristics to be insignificant for the purchase of green cosmetics [15,59,106].

## 5 Conclusion

This study presents an in-depth overview of consumer perceptions of prickly pear seed oil, revealing insights into preferences and purchasing behaviours. Four key consumer segments have been identified - Selective Consumers, Pragmatic Consumers, Sustainable Consumers, and Cautious Consumers - each with distinct priorities and motivations when purchasing prickly pear seed oil. The significance of environmental impact, safety, and labelling information emphasises an increasing ecological awareness and the necessity for transparency and safety. This suggests that sustainable practices and informative labelling are crucial elements for marketing strategies. Factors such as ease of use and appearance, although considered less important, present opportunities for enhancing product appeal. The analysis has revealed that purchasing frequency is significantly influenced by gender and monthly income. Women and higher-income consumers demonstrate a greater propensity to purchase prickly pear seed oil.

It is crucial to acknowledge some limitations of the study. The sampling may not fully encompass the broad range of potential consumers, limiting the generalizability of the findings to a wider population. Additionally, consumer perceptions may be influenced by temporary factors or market trends that were not fully explored in this study. Socio-psychological factors may play a significant role in consumer decisions but were not explored in this context. Finally, an important limitation of this study is that it does not explore in depth the scepticism and confusion surrounding green cosmetics certification, despite it being a crucial issue for the sector. The presence of numerous certification bodies with different standards may generate doubts among consumers about the authenticity and real benefits of certified products, eroding their trust. This represents a gap that future research could fill by examining how transparency of certification and consistency in labelling practices can influence consumer choice. Furthermore, future investigations could explore the role of misleading marketing tactics and greenwashing, which may further exacerbate scepticism towards green cosmetics.

From a theoretical point of view, our study contributes to the body of knowledge on green cosmetics, especially in the context of environmental certifications. It reinforces the idea that consumers are not only attracted by sustainability as such, but also look for informative transparency and tangible reliability in certified products. The results suggest that cosmetics companies should adopt marketing strategies focused on transparency, sustainability and safety. Market segmentation makes it possible to develop customised strategies for each consumer group, thus increasing the effectiveness of advertising campaigns. For example, Selective Consumers might respond better to campaigns focusing on organic and sustainable ingredients, while Pragmatic Consumers might be more sensitive to price and convenience promotions. Finally, from a policy perspective, the results of our study emphasise the need for policies that encourage greater regulation and standardisation of green certifications. Public policies that promote the adoption of sustainable practices and discourage greenwashing could improve consumer confidence and incentivise companies to invest in greener technologies and production processes.

In summary, this study makes an important contribution not only to the producers and sellers of prickly pear seed oil, but also to the green cosmetics field. It highlights how an understanding of different consumer motivations can guide the development of more effective and targeted product and marketing strategies, while at the same time promoting greater ecological awareness among consumers.

## CRediT authorship contribution statement

**Giuseppe Timpanaro:** Writing – review & editing, Writing – original draft, Visualization, Validation, Supervision, Funding acquisition, Conceptualization. **Giulio Cascone:** Writing – original draft, Software, Methodology, Formal analysis, Data curation.

## Ethics and consent

Privacy waivers were granted by each interviewee.

## Data availability statement

The data are available from the corresponding author on reasonable request.

## Funding

This research was funded from the research project "PIACERI: Piano di incentivi per la ricerca di Ateneo 2024/2026" - Principal investigator UNICT prof Salvatore Cosentino, University of Catania.

## Declaration of competing interest

The authors declare that they have no known competing financial interests or personal relationships that could have appeared to influence the work reported in this paper.
