# Supplementary material for: Consumer behavior and sustainability: Exploring Italy's green cosmetics market with prickly pear seed oil
Source: Heliyon. 2025 Jan 25;11(3):e42233. doi: 10.1016/j.heliyon.2025.e42233 (PMC11834030; doi:10.1016/j.heliyon.2025.e42233)
Supplement: Multimedia component 1 [file mmc1.docx]

**Appendix A**

QUESTIONNAIRE ON FACTORS INFLUENCING THE PURCHASE OF PRICKLY PEAR OIL

Among the bio-products that can be extracted from prickly pear fruit is seed oil, a powerful and precious elixir of beauty, known since ancient times and widely used in cosmetic and pharmaceutical preparations.

This will be followed by a short but useful questionnaire to understand whether prickly pear seed oil, with an ethical value (an unwanted waste from the market thus becomes a resource), can find acceptance in your purchasing choices of natural cosmetic oil.

The information will be treated in compliance with the current privacy legislation and with the utmost confidentiality (EU Regulation 2016/679-GDPR). The data collected will be used exclusively for the purposes of developed, non-commercial research.

Section I: ATTRIBUTES OF PRICKLY PEAR OIL

1. Have you ever heard of or used cosmetic vegetable oil or natural cosmetic products?

Yes

No

1. Please, indicate the importance of the following characteristics of prickly pear seed oil using a scale of 1 (unimportant) to 5 (very important):
2. Appearance

☐ 1 ☐ 2 ☐ 3 ☐ 4 ☐ 5

1. Environmental impact

☐ 1 ☐ 2 ☐ 3 ☐ 4 ☐ 5

1. Label information

☐ 1 ☐ 2 ☐ 3 ☐ 4 ☐ 5

1. Brand

☐ 1 ☐ 2 ☐ 3 ☐ 4 ☐ 5

1. Naturalness

☐ 1 ☐ 2 ☐ 3 ☐ 4 ☐ 5

1. Origin

☐ 1 ☐ 2 ☐ 3 ☐ 4 ☐ 5

1. Practicality of use

☐ 1 ☐ 2 ☐ 3 ☐ 4 ☐ 5

1. Price

☐ 1 ☐ 2 ☐ 3 ☐ 4 ☐ 5

1. Safety

☐ 1 ☐ 2 ☐ 3 ☐ 4 ☐ 5

l. Container volume

☐ 1 ☐ 2 ☐ 3 ☐ 4 ☐ 5

1. How often do you buy prickly pear seed oil?

I rarely purchase prickly pear seed oil

I purchase prickly pear seed oil monthly

I purchase prickly pear seed oil weekly

I purchase prickly pear seed oil more than once a week

Section II: PURCHASING HABITS OF PRICKLY PEAR SEED OIL

1. Where do you usually buy prickly pear seed oil?

Online shops (e.g. Amazon)

Perfumery

Pharmacy

Herbalist's shop

Other

1. In which commercial formulation do you prefer prickly pear seed oil?

Pure form

Blended (with argan, sunflower, macadamia, etc.)

1. What color do you prefer for prickly pear seed oil?

Yellow with green undertones

Yellow with red undertones

Yellow with brown undertones

1. Which production technology do you prefer for prickly pear seed oil?

Obtained through fruit maceration

Cold-pressed

Obtained through refining

Section III: SOCIO-DEMOGRAPHIC CHARACTERISTICS

1. Gender

Female

Male

1. Age

19 - 36

37 - 54

> 55

1. Education

Primary

Secondary

High school

University

1. Residence Area

Sicily

Other regions

1. Monthly household income

< 2000 €

2000 - 4000

> 4000
